# Molecular Pathways Involved in Frontotemporal Lobar Degeneration with TDP-43 Proteinopathy: What Can We Learn from Proteomics?

**DOI:** 10.3390/ijms221910298

**Published:** 2021-09-24

**Authors:** Merel O. Mol, Suzanne S. M. Miedema, John C. van Swieten, Jeroen G. J. van Rooij, Elise G. P. Dopper

**Affiliations:** 1 Alzheimer Center, Department of Neurology, Erasmus Medical Center, 3015 GD Rotterdam, The Netherlands; j.c.vanswieten@erasmusmc.nl (J.C.v.S.); j.vanrooij@erasmusmc.nl (J.G.J.v.R.); e.dopper@erasmusmc.nl (E.G.P.D.); 2 Center for Neurogenomics and Cognitive Research, Department of Molecular and Cellular Neurobiology, Amsterdam Neuroscience, Vrije Universiteit Amsterdam, 1081 HV Amsterdam, The Netherlands; s.s.m.miedema@vu.nl

**Keywords:** frontotemporal lobar degeneration, frontotemporal dementia, TDP-43, proteinopathies, proteomics, mass spectrometry

## Abstract

Frontotemporal lobar degeneration (FTLD) is a neurodegenerative disorder clinically characterized by behavioral, language, and motor symptoms, with major impact on the lives of patients and their families. TDP-43 proteinopathy is the underlying neuropathological substrate in the majority of cases, referred to as FTLD-TDP. Several genetic causes have been identified, which have revealed some components of its pathophysiology. However, the exact mechanisms driving FTLD-TDP remain largely unknown, forestalling the development of therapies. Proteomic approaches, in particular high-throughput mass spectrometry, hold promise to help elucidate the pathogenic molecular and cellular alterations. In this review, we describe the main findings of the proteomic profiling studies performed on human FTLD-TDP brain tissue. Subsequently, we address the major biological pathways implicated in FTLD-TDP, by reviewing these data together with knowledge derived from genomic and transcriptomic literature. We illustrate that an integrated perspective, encompassing both proteomic, genetic, and transcriptomic discoveries, is vital to unravel core disease processes, and to enable the identification of disease biomarkers and therapeutic targets for this devastating disorder.

## 1. Introduction

Frontotemporal lobar degeneration (FTLD) is widely known as a severely debilitating and progressive neurodegenerative disorder, clinically characterized by variable degrees of behavioral, language and motor symptoms, collectively named frontotemporal dementia (FTD) [1]. The pathological hallmark of FTLD is atrophy of the frontal and temporal lobes with accumulation of protein aggregates in the cytoplasm or nuclei of neuronal and glial cells [2]. Based on the deposited protein, cases are classified into different molecular subgroups [3]. The largest group (~50%) encompasses FTLD-TDP, with ubiquitin-positive inclusions containing aggregated and hyperphosphorylated TAR DNA binding protein 43 (TDP-43), which can be further classified in FTLD-TDP subtypes A to E, based on histopathological patterns [4]. The topographical distribution of atrophy and TDP-43 pathology defines the clinical FTD phenotypes. Usually, the pathology begins in the prefrontal cortex and amygdala, from where it spreads to other areas, including hippocampus, subcortical regions, brain stem, and spinal cord [5].

Though the majority of FTLD-TDP cases are sporadic, around 10–20% are estimated to be caused by mutations in *GRN* or the *C9orf72* repeat expansion [6]. In addition to these major genetic causes, many other genes have been implicated in FTLD-TDP, including *TARDBP* (encoding TDP-43), *TBK1*, *OPTN*, *VCP*, and *SQSTM1* [6]. These genes have all been linked to both FTLD and amyotrophic lateral sclerosis (ALS), a motor neuron disorder also associated with TDP-43 pathology. Furthermore, genome-wide association studies (GWAS) have revealed *TMEM106B* as important risk modifier, modulating age at onset and disease penetrance in *GRN* mutation carriers [7,8,9]. Importantly, some genetic mutations are correlated to specific pathological features, e.g., *GRN* mutations nearly always result in FTLD-TDP type A with abundant intranuclear inclusions, whereas *C9orf72* is usually associated with type B pathology, showing diffuse cytoplasmic inclusions [4].

Besides the importance for genetic counselling in clinical practice, all genetic discoveries combined have provided a wealth of information regarding the molecular pathways involved in the pathogenesis of FTLD. However, the great difficulty lies in understanding the precise mechanisms that connect these genes to the observed neuropathology with accumulated protein within inclusions. Resolving whether these inclusions are directly related to specific genetic defects, or a symptom of imbalanced protein homeostasis is essential to understand true disease mechanisms, and for the development of disease modifying treatments.

A valuable tool that has developed greatly over the last decade is mass spectrometry technology, which enables comprehensive analysis of protein depositions and aberrant protein expression in the disease-affected brain [10]. In addition to genomic and transcriptomic studies, investigating the proteomic changes can help to unravel disease processes. This is exemplified by a family with a rare hereditary neurodegenerative disorder, where a combination of proteomic analysis of neuronal inclusions and exome sequencing revealed altered functioning of protein kinase A due to a novel *PRKAR1B* mutation as the underlying disease mechanism [11].

In this review, we first introduce proteomic profiling technology and some of the possibilities regarding study designs, after which we describe the main findings of proteomic profiling studies performed on human FTLD-TDP brain tissue. Subsequently, we evaluate how the results relate to genetic discoveries, and to which extent both fields interconnect in unraveling the core molecular pathways involved in FTLD-TDP.

## 2. Proteomic Methodology

### 2.1. Proteomic Profiling Using Mass Spectrometry

A multitude of techniques are currently available to study the proteome. These can be grouped roughly as structural proteomics, functional proteomics, and proteomic profiling. The first two are mostly targeted and hypothesis-driven, aiming to map out the structure and activity of specific proteins or protein complexes [12]. In contrast, proteomic profiling is usually performed in a high-throughput manner by examining thousands of proteins at once, providing a resource for subsequent targeted studies [13]. This approach is widely applied in many different research areas, with mass spectrometry (MS) as the current method of choice. The MS workflow consists of three principal steps: (1) sample preparation including dissection, cell lysis, and protein extraction; (2) protein digestion and separation, where peptides are usually fractionated by liquid chromatography based on their hydrophobicity; and (3) protein identification and quantitation by MS, using either peptide labeling or a label-free strategy [12]. MS techniques are constantly improving, as illustrated by the current transition from traditional data-dependent acquisition (DDA; limited number of precursor peptides are semi-randomly selected) towards data-independent acquisition (DIA; identification of nearly all detectable peptides within a selected mass range), resulting in higher sensitivity and protein coverage [14]. For further technical details and examples in relation to neurodegeneration, we refer to a number of recent reviews [15,16,17,18].

### 2.2. Sample Selection and Processing

Proteomic profiling of different types of substrates, i.e., cells, tissue, or body fluids, can offer unique and complementary insights into disease processes. The approach is dependent on the specific research question and sample availability. In different types of dementia, numerous studies have analyzed plasma, serum, and cerebrospinal fluid (CSF) protein levels to identify potential biomarkers [19,20]. These biofluids are easily accessible during life, but less informative with regard to the potential to uncover the complex molecular changes underlying brain pathology when compared to brain tissue. Proteomics of whole human brain tissue can be challenging due to atrophy affecting protein abundances, differences in cellular composition, and inter- and intra-regional variability of pathology throughout the brain, and during the course of disease progression [21]. These aspects are crucial to bear in mind when designing and interpreting proteomic studies.

The majority of studies up to date use bulk tissue, where sample homogenization results in a mixture of cell types. Some studies correct for this using cell-type specific markers obtained from single-cell profiling, either using a defined set of marker genes, or by enrichment methods using all known genes expressed within a cell type [22,23]. As an alternative to bulk tissue, laser capture microdissection (LCM) is used to isolate specific cell populations [24,25]. This enables the evaluation of particular neuropathological features or vulnerable brain regions, e.g., protein inclusions in FTLD, and amyloid plaques and neurofibrillary tangles in Alzheimer’s disease (AD) [26,27,28]. Next to LCM, various cell sorting and fractionation techniques can be applied to examine specific cells or subcellular compartments such as synaptosomes, mitochondria, or nuclei [29].

Another essential consideration when studying proteinopathies is the lysis method used for sample preparation. Insoluble proteins or protein complexes may not be detected without employing a particular lysis method [30]. Several techniques have been developed to specifically study the protein composition of insoluble plaques and tangles in AD, or neuronal protein aggregates in FTLD [31,32]. Furthermore, the insoluble fractions are often modified by ubiquitination and/or phosphorylation (e.g., aberrant tau phosphorylation in AD), suggesting a role of posttranslational modifications (PTM) in the disease pathogenesis. Proteomic workflows have extended to identification and quantification of PTMs using different approaches [33]. A challenge in this area is the postmortem interval, during which the level of phosphorylated proteins may decrease due to different influences such as hypoxia [34]. Nonetheless, several studies have detected changes in the PTM profile possibly implicated in neurodegenerative processes [35].

### 2.3. Downstream Proteomic Data Analysis

The complex proteomic data, with often hundreds or thousands of proteins identified, necessitates a tailored downstream analysis to filter out information meeting the specific aim of the study. Various software packages and tools are currently available to perform standardized bioinformatics analyses [36]. Describing all possible analytic strategies falls outside the scope of this review. However, we will outline several relevant and widely used methods. For example, network-based approaches are increasingly used to organize the data into unbiased groups or so-called modules of proteins that are co-expressed [37]. The proteins most central to a module are referred to as ‘hubs’. Protein levels in such modules can be compared between groups or correlated with clinical measures. The modules can also be analyzed for biological enrichment using reference databases, including functional pathways, cell types, cellular localization, and genetic risk factors. These module-enrichment profiles can offer important insight into the proteomic composition and pathogenic mechanisms, and comparison of studies on a module level is often more feasible than on protein level, as illustrated by several network analyses of AD brains [18].

Another recent trend is to build protein networks based on known interactions or functional relationships between proteins and their binding partners. These interactions, aggregated in online databases, can be derived from different sources, e.g., experimental data, co-expression, or co-occurrence of proteins in certain tissues/cellular compartments. The resulting protein-protein interaction or signaling networks can facilitate the mechanistic understanding of biological processes and aid in the discovery of functional protein complexes. An important disadvantage is that network-based approaches are generally less suitable to pinpoint more subtle cellular alterations and the roles of unique proteins. Therefore, discovery-driven proteomics is usually followed up by targeted structural or functional studies to deduce the exact molecular mechanisms.

## 3. Proteomic Studies in FTLD-TDP

Table 1 lists the main findings of the nine unbiased (DIA) proteomic studies conducted on FTLD-TDP brain tissue, in comparison to healthy and/or disease controls, so far. The number of proteins with differential abundance across groups varied widely (range 6–407), depending on study design. Five out of nine studies analyzed bulk tissue, with the frontal cortex as most frequently studied region. The other four studies investigated a specific isolated region or cellular inclusions. A label-free MS approach was nearly always the method of choice, albeit using different determination and normalization procedures (e.g., spectral counts, MaxLFQ algorithm). Irrespective of method and region, a total of 95 proteins (15% of the total 640 unique proteins identified) overlapped across the nine studies (Table S1).

### 3.1. Bulk Tissue

The largest study by Umoh et al. compared the protein expression profile of 10 healthy controls with 12 FTLD-TDP, 19 ALS, and 10 ALS-FTD cases, including a subset of 11 ALS cases with C9orf72 repeat expansion [38]. Over 400 unique proteins were determined to be significantly altered among pairwise comparisons. Weighted co-expression network analysis (WGCNA) identified 15 distinct modules of proteins, of which seven differed significantly in FTLD-TDP compared to controls and ALS. Two of these modules were enriched for RNA binding (e.g., CSRP1 as hub protein) and inflammation (e.g., HEPACAM, MSN, GFAP), and showed strong correlation with cognitive dysfunction and TDP-43 pathological burden. Microglial proteins were overrepresented in both modules, suggesting a connection between RNA binding protein dysfunction and microglial activation. Two astrocytic proteins (GFAP, HEPACAM) and two microglial proteins (MSN, TPP1) were selected for validation, and confirmed to be increased in FTLD by immunoblotting and immunostaining, as compared to ALS and controls. Modules enriched for synaptic (e.g., SYT1, DLG4), neuronal (e.g., MAP1B, RTN4), and mitochondrial proteins (e.g., NDUFA2, UQCRC1, and ATP5A1) showed decreased expression in FTLD-TDP, which is possibly partially on account of neuronal loss, and therefore difficult to distinguish from initiating disease processes. The overall expression profile of ALS was similar to controls, while ALS-FTD cases were distributed between FTLD-TDP and ALS without dementia, supporting the notion that proteomic changes in cortical brain tissue define the clinical phenotype within the ALS-FTD spectrum. Altogether, by a systems biology approach this study described the broad proteomic signature of FTLD-TDP. By focusing on protein modules, however, the reported biological processes are relatively broad. Thus, this work can be regarded as a resource for further investigation of more specific molecular changes.

Andrés-Benito et al. specifically analyzed the frontal cortex of 19 *C9orf72* expansion-positive FTLD cases compared to 14 controls, combined with transcriptomics [39]. The biological functions of the 130 deregulated proteins were assessed using Ingenuity Pathway Analysis (IPA) software, which revealed abnormal expression of proteins in FTLD involved in inflammation (e.g., MSN, OPTN), along with several other deregulated pathways related to apoptosis (e.g., PPT1, CTSD), mitochondria (e.g., SOD2), and endocytosis (e.g., ANXA1/5, RAB7A, CLTC). In addition, the authors performed immunoblotting of TDP-43 and C9orf72, and found elevated levels of TDP-43 protein and the C9orf72 short isoform in cases compared to controls. Downregulation of the synaptic protein SNAP25 was also confirmed by immunoblotting. Unfortunately, the authors did not perform a similar validation for other upregulated proteins. The proteomic changes correlated poorly to gene expression, a phenomenon that is often encountered [46]. The authors built an interactome network using IPA, which indicated deregulation of cross-linkers between transcriptional processes and protein degradation mechanisms. This approach illustrates the value of bioinformatics tools to interconnect DNA/RNA regulation to the proteome, though the study also shows that combining the results of different ‘-omics’ can be highly challenging.

Based on the hypothesis of partly overlapping molecular changes, Iridoy et al. compared proteomic changes in the spinal cord between eight healthy controls, eight FTLD-TDP, and nine ALS cases. As this region is generally more affected in ALS, the number of aberrantly expressed proteins was higher in ALS (*n* = 281) versus FTLD (*n* = 52) [40]. Unfortunately, no ALS-FTD cases were included, which might have shown an intermediate profile. The total of 33 overlapping proteins with altered expression in both ALS and FTD compared to controls indicated mitochondrial dysfunction as most significantly enriched for both disorders (e.g., CHCHD3/6, NDUFA2, ATP5I). Immunoblotting confirmed downregulation of the Prohibitin complex proteins (PHB1 and PHB2)—located at the inner mitochondrial membrane—in spinal cord of both ALS and FTLD, but in frontal cortex of FTLD cases only, implying mitochondrial imbalance in both disorders, but with regional differences. By choosing to focus on the overlap between ALS and FTLD, additional deregulated proteins and pathways in FTLD-TDP were not as thoroughly analyzed in this study.

Lachén-Montes et al. attempted to analyze the proteome of the olfactory bulb in a small set of FTLD-TDP cases (*n* = 4), progressive supranuclear palsy (PSP) (*n* = 4) and controls (*n* = 4), despite the fact that this region is not commonly affected in FTLD [41]. Using isobaric labeling with tandem mass tags (TMT), MS indicated only 28 proteins (1% of total quantitated proteome) with differential expression in FTLD and 25 in PSP (one protein overlapping) compared to controls, hindering a satisfactory description of specifically affected pathways. Nonetheless, the authors suggest disruption of vesicle trafficking (RAB3C downregulation), and overproduction of specific cytoskeletal proteins (type VI collagens) in the olfactory bulb in FTLD, though these proteins were not selected for validation by another method.

### 3.2. Isolated Tissue or Cellular Inclusions

The study by Gozal et al. applied LCM in three FTLD-TDP cases and three controls, in order to isolate a specific region of interest, the hippocampal dentate gyrus [42]. Although this comparison revealed 73 dysregulated proteins associated with several biological pathways, the authors solely focused on two cytoskeletal septin proteins, SEPT3 and SEPT7, showing high enrichment in FTLD. Upregulation of these proteins was confirmed by immunoblotting. However, immunohistochemistry using antibodies for these proteins did not overlap with the staining pattern of TDP-43 aggregates. This led the authors to conclude that they mainly detected soluble, non-aggregated proteins in their proteomic data set. In line with this observation, the majority of studies did not report aberrant TDP-43 expression, probably because its pathogenic and aggregated form is difficult to detect.

Therefore, specifically analyzing the pathologic inclusion proteins in the frontal cortex, by using different techniques to yield the detergent-insoluble fractions, gained attention. To reduce variation, in a second study Gozal et al. chose to perform two independent MS strategies on insoluble protein extracts derived by biochemical fractionation [43]. First, 10 FTLD-TDP cases, 10 AD cases, and 10 healthy controls were included in a label-free MS setup, which yielded 50 dysregulated proteins in FTLD compared to AD and control extracts. Second, a labeled approach using isotope tags was performed on protein lysates from four FTLD and four control cases, resulting in 194 proteins altered in FTLD. The fact that only 10 proteins overlapped across both methods illustrates the high variability in proteomic results. Of these 10 proteins, SEPT11 was consistently enriched in FTLD but not in AD, and its upregulation was confirmed by both targeted MS and immunoblotting in four cases. Immunohistochemistry with SEPT11 antibodies did not show overlap with TDP-43 inclusions, but revealed irregular fibrillary structures in the superficial cortical layers in approximately half of the cases. The authors speculated that changes in SEPT11 might affect cytoskeletal function and that its cleaved fragments could result in septin aggregation and cytotoxicity.

Following the same lysis method, Seyfried et al. used an MS quantitation method with labeling of amino acids in cell culture (SILAC) [32]. This approach is thought to enhance the ability to quantify low abundant proteins. Of the 21 proteins showing differential abundance in four FTLD cases versus four controls, the authors further examined the RNA binding protein PSF because of its function in ribonuclear protein (RNP) complexes [47]. Immunoblotting and immunohistochemistry validated enrichment of PSF and truncated fragments in a subset of cases. Interestingly, cytoplasmic accumulation was observed in oligodendrocytes predominantly, possibly reflecting its involvement in white matter changes and neuroinflammation, also seen in FTLD.

Laferrière et al. provided valuable insight into the composition of TDP-43 aggregates, and is so far the only study that compared different subtypes of FTLD-TDP. The authors developed a novel method (SarkoSpin) to purify pathological TDP-43 and other aggregated insoluble proteins from frontal cortical brain samples of FTLD-TDP subtypes A (*n* = 6), B (*n* = 3), C (*n* = 6), ALS cases (*n* = 6), and controls (*n* = 6) [44]. Label-free quantitative MS of the isolated fractions showed that only TDP-43 was consistently enriched in all cases. However, distinct sets of proteins accompanied TDP-43 in ALS, FTLD-TDP type A and type C, possibly underlying subtype specific mechanisms. The associated pathways of the identified proteins are diverse, including mitochondrial (e.g., PDHA1), lysosomal (e.g., ASAH1), neuroinflammation (e.g., HEPACAM), and RNA metabolism (e.g., HNRNPA1). Immunoblotting of a candidate protein for each disease group (ASAH1 for FTLD-TDP type A and TXNL1 for type C) confirmed their enrichment, which correlated with TDP-43 levels in the respective disease subtype. Immunofluorescence of these proteins did not show co-localization with TDP-43. This suggests that these proteins do not directly aggregate with TDP-43, but become insoluble via other, still unexplained, disease mechanisms.

### 3.3. Phosphoproteomics

Herskowitz et al. specifically analyzed the phosphoproteome in the frontal cortex of four FTLD-TDP cases compared to four controls, using MS preceded by phosphopeptide enrichment (IMAC) [45]. Label-free quantitation revealed only six proteins with significantly altered phosphorylation patterns in FTLD, including an increase of NDRG2 and GFAP phosphorylation. These proteins are both highly expressed in astrocytes, suggesting their involvement in reactive astrocytosis in FTLD. The other four proteins with decreased phosphorylation status were not validated by immunoblot analyses and could therefore also reflect overall decreased protein levels.

### 3.4. Fluid Substrates

Though this review focuses on brain tissue proteomics, relevant to mention is that numerous studies in the field of FTD have measured proteins in biological fluids such as plasma, serum, and CSF. Using a variety of techniques, most studies have focused on finding specific diagnostic biomarkers to differentiate FTD from other dementias or neuropsychiatric disorders, and to distinguish specific subtypes or disease stages. A recent review has summarized the most important findings regarding FTD biomarkers [20]. Besides targeted studies, unbiased MS approaches have attempted to disclose disease-associated proteins in CSF. So far, multiple markers have been identified associated with a range of pathways including neuroinflammation (e.g., GFAP, YKL40) [48,49], lysosomal function (e.g., CTSD) [50,51], and synaptic transmission (e.g., SNAP25, SYT1, NPTXR). [52,53].

## 4. Converging Molecular Pathways in FTLD-TDP

**Figure 1:**
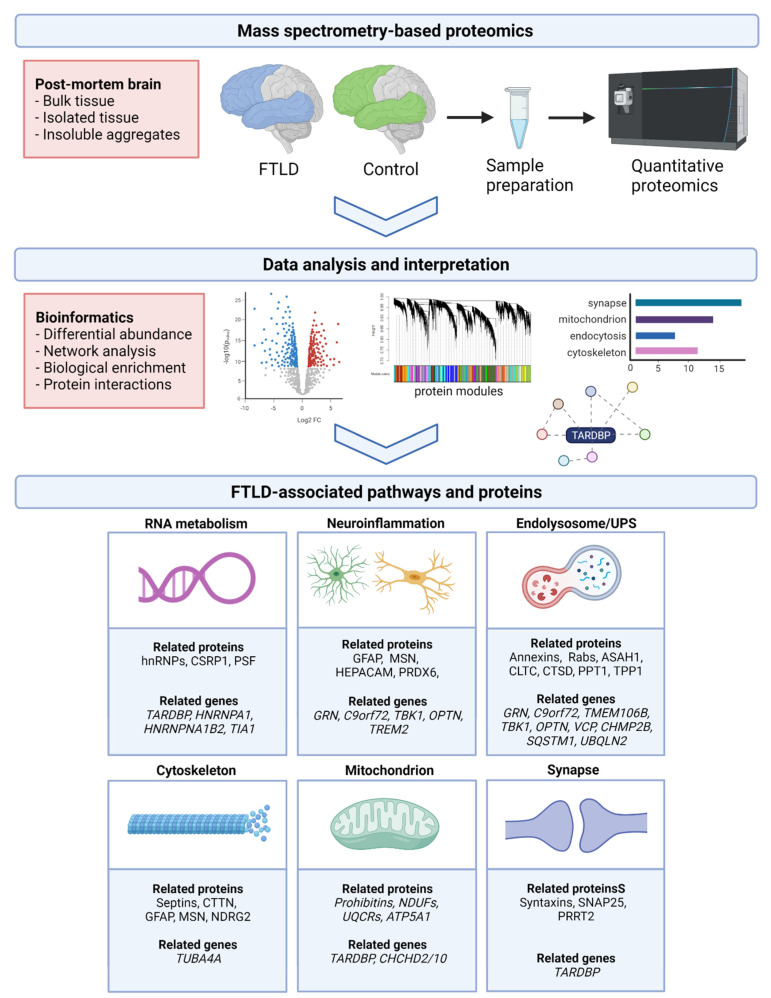
Unbiased proteomics to characterize the proteomic signature of FTLD-TDP. Following brain tissue preparation, several techniques are currently available to quantify the proteomic data generated by mass spectrometry. Different bioinformatics approaches can be applied to analyze the differentially expressed proteome; i.e., to organize these proteins in biologically meaningful groups (modules), to evaluate the protein-protein interactions, or to assess enrichment for specific biological pathways, cell types, and/or genetic risk factors. By reviewing the various proteomic studies performed in FTLD-TDP, six core pathways could be identified that are implicated in the disease pathogenesis. For each pathway, we mention several central proteins/protein families, which were either detected by multiple studies or validated by individual studies (see also Table 2 and Table S1). In addition, the major FTLD genes associated with these pathways are listed, as derived from literature. Importantly, both lists should be regarded as examples and are not exhaustive. *Created with BioRender.com*.

By combining these recent proteomic findings, with knowledge from genetic and transcriptomic studies, we have identified six core pathways implicated in the pathogenesis of FTLD-TDP, which we summarized in the following paragraphs and in Figure 1. Roughly, proteins involved in RNA metabolism, neuroinflammation, endolysosomal system, and cytoskeleton are upregulated, whereas mitochondrial and synaptic proteins are usually decreased in the FTLD-TDP proteome.

Table 2 shows which proteomic studies identified proteins related to each of these pathways, including the most important overlapping proteins/protein families (for a complete list we refer to Table S1), and proteins validated as altered in FTLD-TDP by individual studies.

### 4.1. RNA Metabolism

TDP-43 itself is an RNA binding protein that controls the levels and splicing patterns of hundreds of RNAs [54], implying a possible role for impaired RNA metabolism in the FTLD-TDP disease process. This is underlined by increased expression of RNA binding proteins in five different studies (Table 1 and Table 2). Moreover, mutations in *TARDBP* and several other genes encoding RNA binding proteins (e.g., *hnRNPA1*, *hnRNPA2B1*, *TIA1*) are rare genetic causes of either ALS, FLTD, or both [55,56,57]. This indicates that impaired functioning of these proteins can be at the base of the disease.

The hypothesis regarding their role in neurodegeneration is that neurons are especially vulnerable to disruption of RNA binding protein dosage and dynamics [54,58]. Impaired basal RNA processing may result in imbalanced protein homeostasis due to defects in protein synthesis, nucleocytoplasmic transport, and stress granule dynamics [59,60,61,62]. Moreover, evidence shows that RNA binding proteins may be predisposed to self-promoting aggregation, generating a feed-forward cycle that drives disease progression [63]. Thus, targeting RNA metabolic pathways (e.g., with small molecule drugs interacting with RNA) has been of specific interest in the search for FTLD therapeutic options. However, prioritization of the most promising target is challenging due to the complexity of TDP-43 and its binding partners [62,64,65,66]. Most RNA binding proteins participate in various global cellular pathways, and specifically targeting these proteins is difficult without affecting normal cell metabolism [67]. An important next step is to further characterize candidate proteins involved in initiating disease processes, for example by immunoprecipitation, cross-linking MS [68], or in vitro modeling. After elucidating the specific RNA structures targeted by these proteins, the ultimate goal is to identify organic compounds with drug properties that are able to bind these structures, affecting pathogenic translation patterns, localization, and/or degradation, but without impacting physiological cellular functions.

### 4.2. Neuroinflammation

FTLD-TDP proteomics demonstrated abundant inflammatory proteins with GFAP, HEPACAM, and MSN as central astrocytic and microglial markers as highlighted by six independent studies. Consonant with brain tissue, CSF proteomics has revealed dysregulation of specific features of the inflammatory response including glial cell activation, cytokines, and chemokines [50,69,70]. Especially GFAP, an important cytoskeletal component in mature and developing astrocytes, appears to be a central player. Higher CSF levels of GFAP were previously reported in FTLD as compared to AD and dementia with Lewy bodies [71], and increased serum GFAP correlated with the rate of temporal atrophy in GRN carriers [48]. Its consistent upregulation in the brain, CSF, and blood suggest that this astrocytic protein is a promising biomarker for FTLD. The observed variation in PTMs could function as additional discriminating feature, though this requires evaluation in larger cohorts [45].

The proteomic findings, showing evidence of immune response involvement in FTLD, are in line with knowledge derived from the two major FTLD-TDP genes, *GRN* and *C9orf72* [72,73]. *GRN* encodes progranulin (PGRN), a secreted growth factor which seems to suppress excessive microglial activation, while reduced levels result in the production of pro-inflammatory cytokines [74]. Though the functions of *C9orf72* are somewhat less clear, there is strong evidence for a link to the immune system as several knock out mice studies demonstrated inflammatory phenotypes with severely altered immune responses in macrophages and microglia [75,76,77]. Furthermore, the multifunctional kinase *TBK1* and some of its downstream targets (e.g., *OPTN*), in which loss-of-function mutations were described in FTD-ALS, exert important functions in inflammatory signaling [78,79]. Of interest, Andrés-Benito, et al. found downregulation of OPTN in the frontal cortex of C9orf72 cases, disclosing broader involvement of this protein in FTLD-TDP [39].

Despite all this evidence pointing towards an important role of the inflammatory response, the precise mechanisms in the pathological cascade of FTLD remain uncertain, possibly shifting from neuroprotective to neurotoxic effects over the course of disease [80]. So far, therapeutic approaches aiming at reducing neuroinflammation have not been successful, and dissecting disease specific changes remains challenging. Nonetheless, inflammation and immunomodulatory agents in FTLD remain the focus of countless research projects, in particular the characterization of microglia-driven changes [81,82]. This will hopefully in the future enable selective targeting of harmful inflammatory changes, without affecting the vital roles of immune cells in the brain.

### 4.3. Endolysosomal System

The endolysosomal system is a complex system involving different components, including endocytosis, vesicle trafficking, autophagy, the ubiquitin-proteasome system (UPS), and apoptotic signaling [83,84]. Proteins implicated in either of these components were identified by seven of the FTLD-TDP proteomic studies (e.g., Annexins, Rabs, PPT1, TPP1, CTSD, CLTC), though their connection to the endolysosomal pathways is not always recognized as such. Several lines of evidence suggest that endolysosomal morphological and functional changes, including autophagy dysfunction and impaired vesicle trafficking, are central players in the FTLD disease process [85,86,87]. In addition, many of the genes and proteins associated with FTLD are known to be involved in the endolysosomal system. Foremost, TDP-43 is known to be crucial for lysosome functioning through its role in lysosomal fusion and by regulation of genes critical for the proper function of autophagy and lysosome pathways [88]. Conversely, excess levels of aggregated and accumulated TDP-43 may directly impair autophagy and lysosome homeostasis [89,90].

PGRN is mostly localized within lysosomes, and its deficiency in *GRN* mutation carriers is thought to cause lysosomal defects in a dose-dependent manner [91,92]. This is most evident by the fact that homozygous mutations lead to neuronal ceroid lipofuscinosis (NCL), a lysosomal storage disease [93]. Accordingly, the lysosomal proteins PPT1, TPP1, and CTSD have been associated with both PGRN deficiency and NCL [94,95], and CTSD co-localizes with TDP-43 in the brain [96]. The increased abundance of these proteins in several proteomic studies implies a more general role in the pathogenesis of FTLD. Their potential as diagnostic biomarker merits further investigation, in particular CTSD, which was also demonstrated to be higher in plasma exosomes of FTD and AD patients compared to controls [51].

C9orf72 also localizes to lysosomes and appears to play an important role in autophagy pathways [88,97,98]. A transcriptomic study of *C9orf72* repeat expansion cases particularly indicated involvement of vesicle-mediated transport [85]. Moreover, C9orf72 was shown to physically interact with several Rabs [99], a family of proteins involved in endolysosomal trafficking. Several Rabs were found dysregulated in different directions across the FTLD-TDP proteome. Other genes implicated in FTLD-TDP, including *CHMP2B*, *VCP*, *SQSTM1*, *UBQLN2*, and *TMEM106B*, are directly involved in autophagy-lysosome pathways [88,100].

Deficits in components of endolysosomal networks might render promising therapeutic targets. In fact, evidence derived from cellular and animal models indicates that stimulating autophagy could alleviate TDP-43 neurotoxicity by reducing the cytoplasmic mislocalization and aggregation of TDP-43 [86,101,102]. Modulation of vesicle trafficking with Rab5 or chemical modulators of Rab5 effectors was shown to rescue neurodegeneration in C9orf72 mouse models [103]. Thus, boosting autophagy, promoting vesicular trafficking, and/or enhancing lysosomal degradation all show therapeutic potential in FTLD.

### 4.4. Cytoskeleton

Similar to microarray studies performed in FTLD-TDP [104,105,106], all proteomic studies of cortical brain tissue showed consistent upregulation of proteins related to the cytoskeleton. Two studies highlighted septins [42,43], ubiquitously expressed cytoskeletal proteins, showing seemingly specific enrichment of SEPT11 in the cortical layers of FTLD and ALS [43]. Septins comprise a family of >30 protein isoforms, playing fundamental roles in the development and physiology of various tissues. Several septins showing high expression in the brain have previously been linked to the pathology of neurodevelopmental and neurodegenerative disorders, including AD [107,108,109].

Cytoskeletal defects are observed in different types of neuronal disorders, though it is not always clear if these defects are causative to or a consequence of neurodegeneration [110]. TDP-43 is known to interact with different components of the cytoskeleton, including actin filaments, neurofilaments, and microtubules [111,112,113]. However, components of the cytoskeleton are rarely linked to genetic FTLD-TDP. Recent reports suggest involvement of *TUBA4A*, encoding the tubulin alpha 4A protein, a major constituent of the microtubules [114,115,116]. *TUBA4A* mutations were previously established as a rare cause of ALS. TUBA4A protein was identified in the TDP-43 aggregates of ALS cases, but not in FTLD [44]. Other proteomic studies did not detect changes in tubulin proteins, although Andrés-Benito et al. reported dysregulation of microtubule dynamics [39].

Additional studies are needed to investigate the role of microtubule functioning in the pathogenesis FTLD-TDP. In other neurodegenerative disease, several therapies acting on the neuronal cytoskeleton have shown promising preliminary results (e.g., microtubule stabilizers), though many potential strategies remain to be explored [117]. Considering their consistent increase in soluble protein extracts, cytoskeletal proteins could be particularly relevant in the search for disease biomarkers in CSF and other biofluids.

### 4.5. Mitochondrial Functioning

Downregulation of several protein families related to mitochondrial functioning in six proteomic studies supports the hypothesis that mitochondria are affected in FTLD-TDP. For example, NDUF, UQCR, and ATP5 synthase proteins all constitute a subunit of one of the complexes that together form the mitochondrial respiratory chain. Other mitochondrial proteins involved in FTLD are CHCHD-containing proteins, which are part of the MICOS complex (mitochondrial inner membrane organizing system) [118,119]. Iridoy et al. reported downregulation of two members of this protein family (CHCHD3 and CHCHD6) in the FTLD spinal cord proteome [40]. Mutations in *CHCHD2* and *CHCHD10* were detected in several FTLD, AD, and Parkinson’s disease patients, implying that mitochondrial dysfunction may be at the origin of disease in some cases. How these proteins relate to neurodegenerative processes remains largely unresolved, though a relationship with mitophagy has been suggested [119,120].

TDP-43 is known to be directly related to mitochondrial functioning, and abnormal aggregation of TDP-43 can damage mitochondria and disrupt mitophagy [121,122]. Moreover, an immunoprecipitation study of TDP-43 suggested interactions with several mitochondrial proteins, including PHB2 [123]. As stated before, this protein was shown to be downregulated in the spinal cord of both FTLD and ALS [40]. PHB proteins form a complex in the inner mitochondrial membrane to maintain mitochondrial integrity. This complex illustrates a close interplay between mitochondrial functioning and the autophagy system, through its function as mitophagy receptor involved in targeting damaged or dysfunctional mitochondria for autophagy [124].

In line with cytoskeletal changes, mitochondrial impairment has been implicated in a wide range of neurological disorders likely representing a common risk factor in the development and progression of neurodegeneration [125,126]. Evidence indicates that mitochondrial changes occur early, making it an attractive target for therapy. Indeed, the field of mitochondrial-targeted therapeutics is growing, and different potential candidates have been employed in AD, PD, and ALS [127,128]. Therapeutic approaches aimed at maintaining the proper balance between TDP-43 and mitochondria have been suggested as promising strategy for TDP-43 related disease [122].

### 4.6. Synaptic Functioning

The majority of proteomic studies observed a downregulation of synapse related proteins in FTLD. It must be noted, however, that a decrease of synaptic and neuronal proteins is a commonality across proteomic studies of neurodegenerative disorders, and is often considered to be partially related to tissue degeneration. Nevertheless, a prominent role of TDP-43 and other RNA binding proteins in maintaining synaptic functioning is widely accepted, since many of its RNA targets are involved in synaptic transmission and plasticity [54,62,65,129]. A growing body of evidence suggest that dysfunctional RNA metabolism lies at the root of synaptic loss in TDP-43 related disorders, via the impact of altered RNA granule dynamics [61,130]. Moreover, disease models have shown a direct link between mutant TDP-43 mislocalization and synaptic morphological and functional changes [131]. Since TDP-43 regulates thousands of RNAs, it will be a major challenge to dissect the mechanistic link between RNA metabolism, synaptic dysfunction, and neurodegeneration. Deciphering the precise roles of TDP-43 at the synapse, including identification of target RNAs critical for synaptic function, will be essential before these processes can be considered as therapeutic targets in FTLD.

## 5. Conclusions and Future Directions

Proteomic studies of FTLD-TDP are on the rise and can provide valuable knowledge on the pathogenic mechanisms driving this devastating disorder. An important challenge is to bridge the gap between proteomic and genomic studies, and to deduce the vast amount of data into relevant biological information. Bearing in mind the various substrates and study designs, which evidently result in highly variable findings, we provided an outline of the different proteomic profiling studies performed in in FTLD-TDP. By reviewing the main coincident findings combined with knowledge derived from genetic studies, we considered several core pathways involved in the FTLD-TDP disease process. Some of these pathways show potential to pinpoint candidate proteins as therapeutic targets or diagnostic biomarkers. However, most proteomic studies are relatively small and often do not differentiate between FTLD-TDP subtypes or disease stages. These limitations hinder the recognition of processes critical for disease onset and progression, in the midst of those related to common neurodegeneration.

Future proteomic studies examining larger numbers of cases, ideally comparing different brain regions, disease stages, subtypes of FTLD, and other types of dementias, will help to further unravel disease specific proteomic signatures, and to enhance our understanding of genetic-pathologic correlations. Most informative would be to study affected brain regions without severe degeneration, and to compare the proteomic changes on a cellular level. Encouraging is that single-cell techniques are advancing rapidly, which can help to improve the proteomic profiling of distinct types of neurons and glial cells [132]. This will ultimately form the basis for subsequent targeted investigations, to validate and functionally characterize candidate proteins. However, focusing on unique proteins may come with a caveat, as a holistic view on the various involved pathways and protein-protein interactions seems essential to grasp the complex network of changes leading to FTLD. In line with this, we emphasize the value of combined analyses with plasma, serum, and CSF proteomics, and with other ‘omics’ approaches. We anticipate that integrating these various strategies is critical to fulfill urgent needs such as biomarkers for diagnosis and disease monitoring of FTLD and, ultimately, to pave the way for tailored therapeutic interventions.

## Figures and Tables

**Table 1:** Unbiased proteomic studies of FTLD-TDP brain tissue, including their main findings. Studies are ordered according to study design. All studies included FTLD-TDP cases and healthy controls, and some also included other disease types. A few studies included specific TDP subtypes, but most did not specify the subtype. The major pathways are provided for FTLD-TDP cases as compared to healthy controls, as reported by the studies. Some included both upregulated and downregulated proteins. Abbreviations: FTLD-TDP, frontotemporal lobar degeneration with TDP-43 pathology; DEP, differentially expressed proteins; ALS, amyotrophic lateral sclerosis; PSP, progressive supranuclear palsy; MS, mass spectrometry; TMT, tandem mass tag; SILAC, stable isotope labeling with amino acids in cell culture; CDIT, culture-derived isotope tags; SC, spectral counts; XIC, extracted ion chromatogram; IMAC, immobilized metal affinity chromatography. Progenesis and MaxLFQ are both software packages to perform label-free quantitation. ^a^ This study performed two independent MS strategies, and focused on the overlapping proteins of both datasets. ^b^ A distinct set of proteins was identified for each TDP subtype, but TDP-43 protein was detected in all cases.

Study	Cases, Subtypes	FTLD Cases, N	Healthy Controls, N	Region	Method	DEP in FTLD vs. Controls, N	Upregulated Pathways	Downregulated Pathways
**Bulk tissue**								
Umoh, et al., 2017 [38]	FTLD-TDP; ALS; ALS-FTD	12	10	Frontal cortex	MS, label-free (MaxLFQ)	407	RNA metabolism, neuro-inflammation, homeostatic processes, zinc ion binding	Mitochondrion, synapse, neuronal differentiation
Andrés-Benito, et al., 2019 [39]	FTLD-TDP type B (*C9orf72*)	19	14	Frontal cortex	MS, label-free (Progenesis)	130	Neuroinflammation, apoptosis, metabolism, phagocytosis, endocytosis, oxidative stress	Mitochondrion, synapse, metabolism, microtubules
Iridoy, et al., 2018 [40]	FTLD-TDP; ALS	8	8	Spinal cord	MS, label-free (Progenesis)	52	Mitochondrion, metabolism	Mitochondrion, protein kinase signaling
Lachén-Montes, et al., 2019 [41]	FTLD-TDP; PSP	4	4	Olfactory bulb	MS, TMT	28	Cytoskeleton, apoptosis, protein synthesis	Vesicle trafficking, synapse, protein kinase signaling
**Isolated tissue or cellular inclusions**								
Gozal, et al., 2011 [42]	FTLD-TDP	3	3	Hippocampus, dentate gyrus	MS, label-free (SC)	73	Cytoskeleton, metabolism, oxidative stress, protein degradation	Mitochondrion, metabolism
Gozal, et al., 2011 [43]	FTLD-TDP; AD	10	10	Frontal cortex, inclusions	MS; label-free (XIC) and CDIT ^a^	10 ^a^	Cytoskeleton, glutamate transporter activity, cell adhesion	Synapse, metabolism
Seyfried, et al., 2012 [32]	FTLD-TDP	4	4	Frontal cortex, inclusions	MS; SILAC and label-free (SC)	21	RNA metabolism, cyto-skeleton, mitochondrion, metabolism, endoplasmic reticulum, membrane/transport	-
Laferrière, et al., 2019 [44]	FTLD-TDP types A, B, and C; ALS	15	6	Frontal cortex, inclusions	MS, label-free (SC)	28 ^b^	RNA metabolism, neuro-inflammation. protein degradation, cytoskeleton, mitochondrion, cell adhesion, lysosome	-
**Phosphoproteomics**								
Herszkowitz, et al., 2011 [45]	FTLD-TDP	4	4	Frontal cortex	IMAC; MS, label-free (SC)	6	Cytoskeleton	Microtubule, synapse, chaperones

**Table 2:** Evidence for the involvement of functional pathways in FTLD-TDP following the different proteomic studies. The table indicates which studies reported proteins related to the six main pathways. Note that this overview is based on all proteins reported as significantly altered in FTLD-TDP, and does not always correspond to the pathways highlighted by individual studies (as shown in Table 1). The last two columns provide the major overlapping proteins and protein families, and candidate proteins of individual studies that were confirmed dysregulated in FTLD-TDP by additional validation experiments (e.g., immunoblotting, immunohistochemistry). The proteins of both columns combined are also depicted in Figure 1. A complete list of overlapping proteins can be found in Table S1.

Functional Pathway	Umoh et al. [38]	Andrés- Benito et al. [39]	Iridoy et al. [40]	Lachén-Montes et al. [41]	Gozal et al. [42]	Gozal et al. [43]	Seyfried et al. [32]	Laferrière et al. [44]	Herszk-owitz et al. [45]	Overlapping Proteins /Protein Families	Validated Proteins
RNA metabolism	X	X			X		X	X		hnRNPs, CSRP1	PSF
Neuroinflammation	X	X			X	X		X	X	GFAP, MSN, HEPACAM, PRDX6	GFAP, MSN, HEPACAM
Endolysosomal system	X	X	X	X	X		X	X		Annexins, Rabs, ASAH1, CLTC, CTSD, PPT1	TPP1, ASAH1
Cytoskeleton	X	X	X	X	X	X	X	X	X	Septins, CTTN, GFAP, MSN	SEPT3, SEPT7, SEPT11, NDRG2
Mitochondrial functioning	X	X	X		X		X	X		Prohibitins, NDUFs, UQCRs, ATP5A1,	PHB1, PHB2
Synaptic functioning	X	X	X	X	X	X			X	Syntaxins, SNAP25, PRRT2	SNAP25

## Data Availability

Not applicable.

## Supplementary Materials

Supplementary materials can be found at https://www.mdpi.com/article/10.3390/ijms221910298/s1.

## Abbreviations

ALS	Amyotrophic lateral sclerosis
C9orf72	Chromosome 9 open reading frame 72
CHCHD	coiled-coil-helix-coiled-coil-helix domain
CHMP2B	Charged multivesicular body protein 2B
CSF	Cerebrospinal fluid
CLTC	Clathrin heavy chain
CTSD	Cathepsin D
CSRP1	Cysteine and glycine rich protein 1
CTTN	Cortactin
TDP-43	TAR DNA-binding protein 43
FTD	Frontotemporal dementia
FTLD	Frontotemporal lobar degeneration
GFAP	Glial fibrillary acidic protein
GRN	Granulin precursor
HEPACAM	Hepatic and glial cell adhesion molecule
hnRNP	Heterogeneous ribonuclear protein
LCM	Laser capture microdissection
MAPT	Microtubule associated protein tau
MS	Mass spectrometry
MSN	Moesin
NDRG2	N-myc downstream-regulated gene 2 protein
NDUF	NADH dehydrogenase (ubiquinone) proteins
NPTX1	Neuronal pentraxin 1
OPTN	Optineurin
PGRN	Progranulin
PHB	Prohibitin
PPT1	Palmitoyl-protein thioesterase 1
PRDX6	Peroxiredoxin 6
PSF	PTB-associated splicing factor
PTM	Posttranslational modification
SEPT	Septin
TARDBP	TAR DNA binding protein 43
TBK1	TANK binding kinase 1
TIA1	TIA1 cytotoxic granule associated RNA binding protein
TMEM106B	Transmembrane protein 106B
SILAC	Stable Isotope Labeling with Amino acids in Cell culture
SNAP25	Synaptosome associated protein 25
STXBP1	Syntaxin binding protein 1
TREM2	Triggering receptor expressed on myeloid cells 2
TUBA4A	Tubulin alpha 4a
SQSTM1	Sequestosome 1
UBQLN2	Ubiquilin 2
UPS	Ubiquitin-proteasome system
UQCR	Ubiquinol-cytochrome C reductase proteins
VCP	Valosin containing protein
